# Roles of Sema4D and Plexin-B1 in tumor progression

**DOI:** 10.1186/1476-4598-9-251

**Published:** 2010-09-21

**Authors:** Ewe Seng Ch'ng, Atsushi Kumanogoh

**Affiliations:** 1 Department of Pathology, School of Medical Sciences, Universiti Sains Malaysia, Malaysia; 2 Department of Immunopathology, Immunology Frontier Research Center, Research Institute for Microbial Diseases, Osaka University, Japan

## Abstract

Sema4D, also known as CD100, is a protein belonging to class IV semaphorin. Its physiologic roles in the immune and nervous systems have been extensively explored. However, the roles of Sema4D have extended beyond these traditionally studied territories. Via interaction with its high affinity receptor Plexin-B1, Sema4D-Plexin-B1 involvement in tumor progression is strongly implied. Here, we critically review and delineate the Sema4D-Plexin-B1 interaction in many facets of tumor progression: tumor angiogenesis, regulation of tumor-associated macrophages and control of invasive growth. We correlate the in vitro and in vivo experimental data with the clinical study outcomes, and present a molecular mechanistic basis accounting for the intriguingly contradicting results from these recent studies.

## 1. Introduction

Semaphorins are a large family of secreted, transmembrane or glycosylphosphatidylinositol-linked proteins containing a phylogenetically conserved extracellular "sema" domain. They are classified into eight classes, of which classes 3 to 7 contain vertebrate semaphorins[1]. Their major receptors have been identified as plexins. In contrast to semaphorins, plexins are transmembrane proteins containing a phylogenetically conserved intracellular "sex and plexins" domain. They are subdivided into plexin A to D subfamilies[2].

Since the discovery of semaphorins as axon guidance cues, semaphorin-plexin interactions have been traditionally best described in the nervous system. Recently their interactions in other systems have also been explored, notably in the cardiovascular development, following revelation of their regulatory roles in cellular motility[3,4]. Intriguingly, semaphorins are also extensively involved in immune response regulation, qualifying some of them as "immune" semaphorins[5]. Recent studies have also focused on the pathological aspects, in particular on their links to several hallmarks of cancer such as tumor angiogenesis and invasiveness. Indeed, these processes recapitulate the many semaphorin-plexin roles in physiologic development. Several recent reviews nicely summarized the accumulating evidence of semaphorin-plexin involvement in cancer progression [6-9]; however, the majority of the content referred to particularly secreted class 3 semaphorins. In this review, we focus on a transmembrane class 4 semaphorin, Sema4D and its receptor, plenxin-B1. We also present a molecular mechanistic basis to reconcile the conflicting results of these molecules in tumor progression from the recent studies.

## 2. Sema4D has versatile roles in immune regulation and axonal guidance

Sema4D, also known as CD100, is a 150kD homodimeric transmembrane glycoprotein first identified in immune cells[10-12], and later classified as a member of class 4 semaphorin based on its distinctive structure[1].

Biological activities of Sema4D are well characterized in the immune system. Sema4D is highly expressed in resting T cells. Although its expression is low in resting B cells and antigen-presenting cells, Sema4D is up-regulated in these cells upon activation. As a ligand acting via its low affinity receptor CD72, Sema4D promotes the aggregation and survival of B cells, and enhances the activation of B cells in antibody production[11,13,14]. Sema4D is also involved in the activation and maturation of antigen-presenting cells[14,15]. Via its high affinity receptor Plexin-B1[2], Sema4D inhibits the migration of monocytic and B-cell lineage cells[16,17]. Reciprocally, acting as a receptor, Sema4D regulates T cell activation[18,19], and is involved in the terminal stages of B cell differentiation[20].

Sema4D is also widely expressed in embryonic nervous tissue, especially in cortical plate and dorsal root ganglia[12], and postnatally in white matter, especially in myelinating oligodendrocytes[21]. As an axonal guidance cue in central nervous tissue, Sema4D functions in a seemingly contradicting fashion. For example, it induces growth cone collapse in primary hippocampal neurons[22,23], but stimulates axonal outgrowth of embryonic dorsal root ganglion neurons[24] and guides the migratory process of gonadotropin hormone-releasing hormone-1 cells[25]. Contradictory to its interaction with CD72 in the immune system, Sema4D interacts with its high affinity receptor, Plexin-B1, in the nervous tissue.

**Figure 1:**
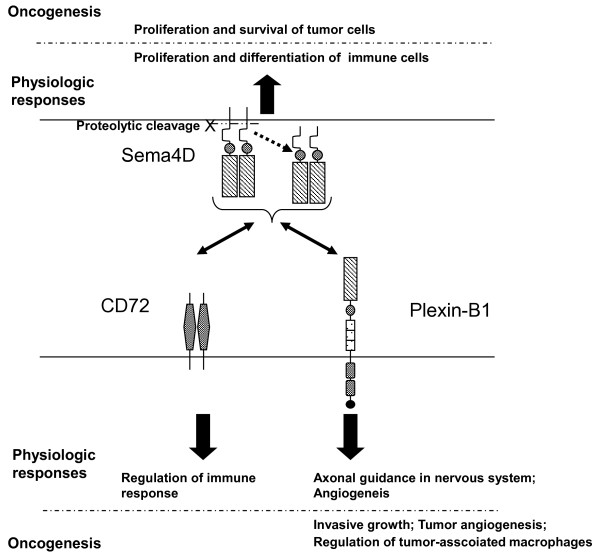
**Interaction of Sema4D and its receptors**. Sema4D interacts with its low and high affinity receptors, CD72 and Plexin-B1, respectively to elicit various physiologic responses as well as oncogenesis.

However, cumulative data have suggested wider roles for Sema4D. In vitro and in vivo studies recently have given many insights into the involvement of Sema4D in tumor progression via interaction with Plexin-B1 (Figure 1). The mechanistic roles of Sema4D and Plexin-B1 in the tumor microenviroment such as tumor angiogenesis, regulation of tumor-associated macrophages as well as the invasiveness of the tumor itself are highlighted in the following discussion.

## 3. Sema4D acts as an angiogenic factor through its high affinity receptor Plexin-B1

Evidence demonstrating the proangiogenic properties of Sema4D has emerged from several in vitro and in vivo studies. Sema4D is able to induce cell migration and promote tubulogenesis in endothelial cells, mimicking pivotal events in angiogenesis. Notably, this angiogenic response elicited by Sema4D is comparable to that by other well-known angiogenic molecules such as VEGF-A, HGF, and bFGF and is independent of any up-regulation of these molecules. In vivo studies employing chick chorioallantoic membrane (CAM) assay and Matigel plug assay confirmed this finding[26,27].

The signaling mechanism behind this Sema4D-induced angiogenic response has also been extensively studied. Plexin-B1, a high affinity receptor for Sema4D, is highly expressed in endothelial cells. Sema4D specifically binds and activates this endothelial receptor to elicit an angiogenic effect[26,27]. However, the signaling cascade beyond the Sema4D-Plexin-B1 interaction is complex and is still debated as described below.

**Figure 2:**
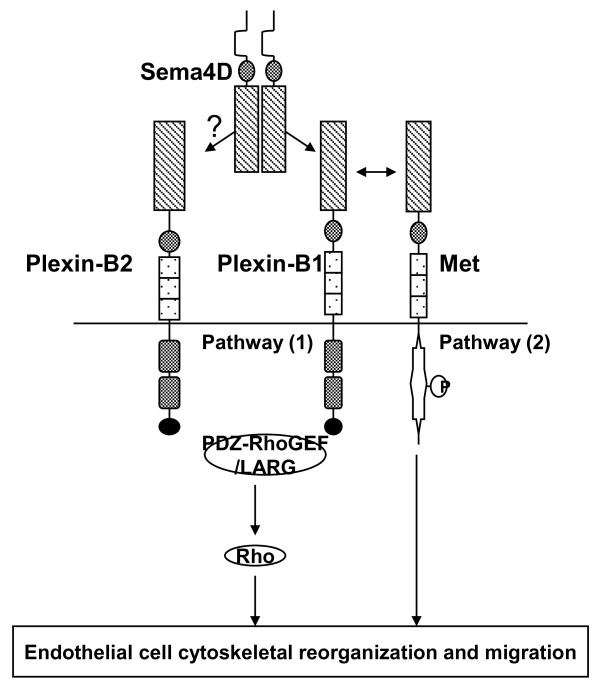
**Sema4D-Plexin-B1 signaling pathways in angiogenesis**. Sema4D engages Plexin-B1 to induce angiogenesis and tumor angiogenesis via two independent downstream pathways. Plexin-B2 might also play a redundant role in this aspect.

Regarding the angiogenic process, two mechanisms have been proposed with conflicting results (Figure 2.). One mechanism proposes a signaling mechanism utilizing the C-terminal PDZ-binding motif of Plexin-B1, which binds two RhoGEFs, PDZ-RhoGEF and LARG, and subsequently activates RhoA. Through the engagement of integrins, this pathway also activates an intracellular tyrosine kinase cascade, notably one that involves Pyk2[27-29]. The other proposed mechanism involves Met activation and tyrosine phosphorylation through Plexin-B1-Met interaction upon Sema4D binding[26]. Both of these mechanisms lead to cytoskeletal reorganization in endothelial cells and their migration.

Given its proangiogenic properties, the possible involvement of Sema4D in tumor angiogenesis was explored. Sema4D is indeed highly expressed in a wide range of human tumors such as prostate, colon, breast, oral, head and neck carcinomas as well as soft tissue sarcomas[30,31]. In one study employing head and neck squamous cell carcinoma cell lines with high expression of Sema4D, tumor growth and tumor angiogenesis in vivo were greatly impaired in the absence of Sema4D[31]. In addition, induction of angiogenesis by tumor cells was made possible by proteolytic cleavage of this membrane-bound Sema4D from the tumor cell surface, releasing its soluble form and thereby permitting Sema4D to act on local as well as distant tumor microenvironments[32].

Another study employing breast cancer cell lines in Sema4D knockout mice demonstrated that the presence of Sema4D in the tumor microenvironment is important for tumor growth and metastasis, which is attributed to tumor vessel maturation induced by Sema4D[33]. Contradicting to the role of tumor-derived Sema4D in head and neck squamous cell carcinoma, this study however showed that tumor-associated macrophages but not tumor cells per se are the main cells that produce Sema4D in the tumor microenvironment, which enhances angiogenesis and contributes to tumor growth[33]. This discrepancy remains unsolved possibly due to differences between these tumor cell lines.

Similar to other in vitro studies, tumor angiogenesis induction by Sema4D is mediated through its interaction with Plexin-B1[31] (Figure 2). A study using Sema4D-expressing melanoma transplants in Plexin-B1 knockout mice, however, showed no significant impairment in tumor angiogenesis. This result indicated that Plexin-B1 is sufficient but not essential for Sema4D-induced tumor angiogenesis; possibly the redundant lower affinity receptors such as Plexin-B2 could be engaged by Sema4D in tumor angiogenesis[34] (Figure 2).

## 4. Sema4D regulates monocytic lineage cells and tumor-associated macrophages in tumor microenvironment

Focusing on the effect of Sema4D on a monocytic lineage, an inhibitory effect on cell migration was demonstrated[16]. A recent study also showed that sequential engagement of CD72 and Plexin-B1 is crucial in this inhibitory effect during the maturation of monocytes into immature dendritic cells. Modulation of cytokine production towards either an anti-inflammatory or a pro-inflammatory cytokine profile seems to be dependent on Sema4D concentration[14,17]. Upon activation, Sema4D expression in dendritic cells is up-regulated. Augmentation of response in an autocrine manner by the proteolytic release of Sema4D acting on receptors in the same cells could ensue[15]. In tumor-associated macrophages, although the lack of Sema4D itself did not alter the differentiation and activation capabilities as well as the cytokine production profile, a significantly lower number of tumor-associated macrophages was observed in the tumor microenvironment[33]. However, the precise mechanism behind these findings awaits elucidation.

Considering these results, the inhibition of cell migration by Sema4D could be alternately interpreted as a means of prolonging cell-cell contact in a physiologic immune response. Exploitation of this mechanism by tumor cells would lead to the retention of tumor-associated macrophages and the subsequent induction of angiogenesis could therefore be triggered.

## 5. Sema4D-Plexin-B1 interaction controls invasive growth

**Figure 3:**
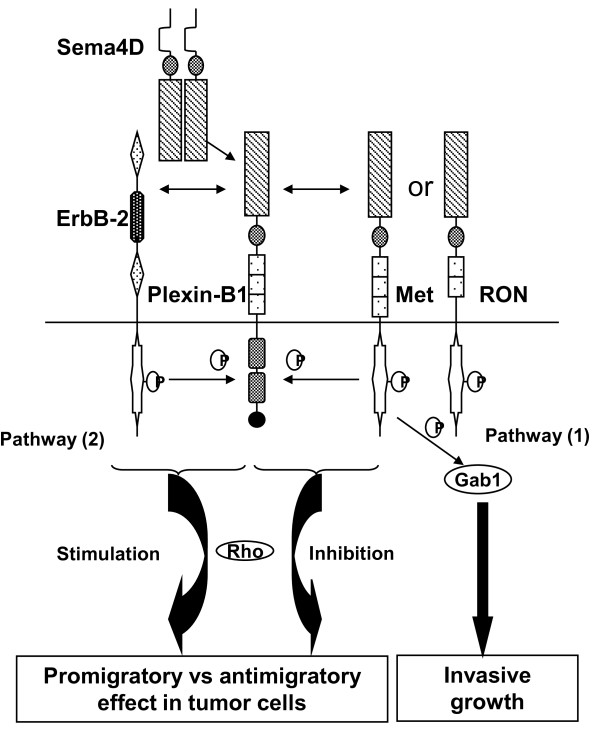
**Control of cellular motility by Sema 4D-Plexin-B1 interation via the coopeative mechanism**. Coupled with the Met, Ron or ErbB-2 tyrosine kinase receptor at the cellular membrane surface, Sema4D interacts with Plexin-B1 to control cellular motility and invasive growth via Gab1 or Rho-dependant pathways.

Invasive growth is a complex morphogenetic program that includes several steps such as cell-cell dissociation, motility, colonization of new sites, proliferation and survival. Invasive growth under physiologic conditions occurs during embryogenesis and wound healing. However, when this program is hijacked, cancer progression and metastasis, the pathologic counterpart, ensue[35]. An invasive growth phenotype in epithelial cells could be elicited by Sema4D through Plexin-B1 coupled with the Met signaling pathway[36]. Another study along the same line showed that Ron, a scatter factor receptor similar to Met, also couples with Plexin-B1 in transducing Sema4D signals (Figure 3). Moreover, over-expression of Plexin-B1 is observed in breast, colon, liver, pancreatic and gastric carcinoma cell lines. Signaling through Plexin-B1 coupled with Met is essential for invasive response in these cell lines[26,37,38].

However, signaling by Sema4D through Plexin-B1 coupling with Met in invasive response is partially disputed by a recent study. This study demonstrated that the antimigratory or promigratory effects of Sema4D in breast cancer cell lines depend on the stoichiometry of the expression of Plexin-B1, Met and ErbB-2 [39]. ErbB-2 is a well-known poor prognostic factor in breast cancer. Coupling of Plexin-B1 with Met inhibits migration, while coupling with ErbB-2 promotes it. By competing for Plexin-B1, these two molecules counter each other's effects in the same tumor cell, determining the net antimigratory or promigratory effect of Sema4D. These molecular complexes further control a downstream Rho-dependent pathway that is similar to the mechanism proposed to promote endothelial cell angiogenesis[39,40] (Figure 3).

**Figure 4:**
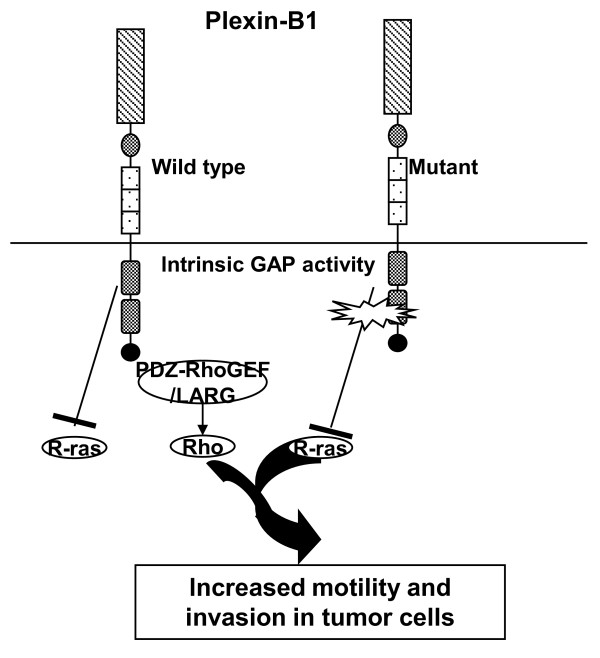
**Control of cellular motility by Sema 4D-Plexin-B1 interation via the intrinsic mechanism**. Decreased intrinsic GAP activity of Plexin-B1 also leads to preferential signaling through the stimulatory R-Ras and Rho-dependant pathways.

Although not shown in most studies involving cancer cell lines, another line of investigation of Sema4D and Plexin-B1 interactions centered on the collapse of neurite outgrowth. Recently, Plexin-B1 has been discovered to have intrinsic GTPase-activating protein (GAP) activity towards R-Ras[23]. Inactivation of R-Ras reduces cell adhesion, cell migration, and growth cone outgrowth[41]. In contrast to the activating pathways discussed above, this inhibitory pathway is believed to inhibit cancer cell migration. Mutations that abolish the R-Ras GAP activity of Plexin-B1 in cell lines promote cell adhesion, motility and invasion[42] (Figure 4).

## 6. High expression of Sema4D is a poor prognostic factor in soft tissue sarcomas

Cumulative evidence regarding the roles of Sema4D- Plexin-B1 interaction in tumor progression came from in vitro and in vivo studies. A recent clinical study on soft tissue sarcomas revealed that higher Sema4D expression was correlated with higher mitotic counts, cellularity, a higher necrosis ratio and Ki-67 index[30], suggesting a proliferative advantage in tumors with higher Sema4D expression. Accordingly, higher Sema4D expression is associated with poorer overall survival and disease-free survival[30] (Table 1). Similarly, via activation by Plexin-B1, Sema4D-expressing CD38+ B-cell chronic lymphocytic leukemic cells have better viability and a higher proliferative rate[43].

**Table 1:** Important findings regarding expression of Sema4D and Plexin-B1 in various human cancers

Cancer Type	Findings	Prognosis
Soft tissue sarcomas[30]	Higher Sema4D expression was associated with higher mitotic counts, cellularity, necrotic ratio and Ki-67 index	Poorer overall survival and disease-free survival with higher Sema4D expression

B-cell chronic lymphocytic leukemia[43]	Sema4D-expressing CD38+ B-CLL cells showed increased proliferation and survival.	Not available

Breast carcinoma[44,45]	Lower Plexin-B1 was associated with a higher histologic grade, proliferative index and ErbB-2 expression in estrogen receptor-positive cases	Poorer disease-free survival with lower Plexin-B1 expression in estrogen receptor- positive cases

Renal cell carcinoma[46]	81% of renal cell carcinoma lost the plexin-B1 expression	Not available

Breast and Ovarian Carcinomas[47]	Co-expression of Plexin-B1 and Met resulted in Met activation and was associated with a higher tumor grade and lymph node metastasis	Not available

Prostate adenocarcinoma [42]	Expression of Plexin-B1 and Sema4D was significantly correlated and both were highly expressed in cancer cases (77% and 58% respectively)	Not available

## 7. Clinical studies offer insights into the role of Plexin-B1 in human oncogenesis

Recently clinical studies have also provided further insights into the significance of Plexin-B1 in human cancers (Table 1.). In human breast cancers, gene expression microarray analysis showed that low Plexin-B1 expression was associated with high histologic grading, ErbB-2 over-expression and a high proliferative index in estrogen receptor-positive breast cancers. Low Plexin-B1 expression also predicts poor outcome in terms of disease-free survival in estrogen receptor-positive breast cancers[44,45]. Similarly, Plexin-B1 expression is also down-regulated in renal cell carcinomas where 169 out of 209 carcinomas showed negativity in a recent report. Reintroducing Plexin-B1 into the renal adenocarcinoma cell line reduced the proliferative rate[46]. These results imply a tumor suppressor role of Plexin-B1 in oncogenesis.

In contrast, a recent study on human breast and ovarian cancers using immunohistochemistry showed that co-expression of Plexin-B1 and Met was significantly associated with a higher tumor grade and lymph node metastasis. Met was activated in the presence of Plexin-B1 expression in a majority of these cancers[47]. In human prostate cancers, Plexin-B1 and Sema4D are strongly associated and highly expressed compared to the non-neoplastic tissue (77% versus 6% and 58% versus 3.5% respectively). Mutations in Plexin-B1 are frequently detected in primary and metastatic prostate cancers[42]. Among the mutations, the more frequent amino acid changes are mapped to the Rho GTPase binding domain of Plexin-B1. Structural analysis revealed a reduced Rho GTPase binding affinity in some of these mutations[48]. In vitro studies showed that these mutations impaired the R-RasGAP activity of Plexin-B1, leading to increased cell adhesion, motility and invasion[42]. These results instead suggest an oncogenic nature of Plexin-B1. Interestingly, a recent study on melanoma also revealed contradicting effects of Plexin-B1 expression on cell migration: enhancement in primary tumor cells but inhibition in metastatic tumor cells[49].

## 8. Intrinsic and cooperative mechanisms determine the overall signaling of Plexin-B1 in tumor progression

Recognizing the fact that both the stimulatory and inhibitory domains coexist in the Plexin-B1 cytoplasmic domain, we speculate that "intra-molecular" preferential Plexin-B1 signaling pathways in cancers could constitute a sound explanation to reconcile these conflicting clinical results of Plexin-B1 in tumor progression. Inhibitory signals of Plexin-B1 would enable it to function as a tumor suppressor gene as implied from the breast and renal cancer studies[44-46]. In contrast, impairment in the Plexin-B1 inhibitory pathway due to mutation, together with over-expression of Plexin-B1 would allow its stimulatory signals to prevail and act as an oncogene such as in prostate cancer[42].

In addition, fine-tuning the inhibitory or stimulatory downstream signaling of Plexin-B1 is possible through the association with competing receptor tyrosine kinases such as Met and ErbB-2, which in turn would deliver additional signals. Although a recent study on human breast and ovarian cancers implies that Plexin-B1 functions as an oncogene when coupled with Met activation[47], involvement of ErbB-2 in this association has yet to be addressed in clinical studies. The available in vitro data, nonetheless, support the ErbB-2 instead of Met coupling in determining the invasiveness of tumor cell lines[39,40].

## Conclusion and perspective

Although the interaction of Sema4D and Plexin-B1 has profound physiologic effects in the immune and nervous systems, emerging data suggests their involvement in tumor progression and especially in tumor angiogenesis where Sema4D acts as a proangiogenic factor via Plexin-B1. Over-expression of Sema4D in tumor confers on the tumor cells a selective proliferative advantage. Sema4D might also influence via Plexin-B1 several functional aspects of tumor-associated macrophages such as their migratory capability and capability to induce tumor angiogenesis. In contrast to responses elicited in non-tumorous components such as endothelial cells and macrophages, signaling through Plexin-B1 in various cancers gives rise to seemingly contradicting responses, as highlighted in this review. Current results suggest that either the stimulatory or inhibitory signaling could be induced via the respective stimulatory or inhibitory domain in the Plexin-B1 molecule itself. This could also be achieved via association with the competing receptor tyrosine kinases such as Met and ErbB-2 (Figure 3 and 4). This double-checkpoint mechanism based on intrinsic and cooperative signaling signifies the complexity and importance of Plexin-B1 signaling in tumor progression. Further studies will certainly solidify the basis for these unique mechanisms.

## List of abbreviations

VEGF: vascular endothelial growth factor; HGF: hepatocyte growth factor; bFGF: basic fibroblast growth factor; PDZ: PSD-95/Dlg/ZO-1; GEF: guanine nucleotide exchange factor; LARG: leukemia-associated Rho GEF; Pyk2: Proline-rich tyrosine kinase 2; MET: mesenchymal-epithelial transition factor; Ron: Recepteur d'origine Nantais; GAP: GTPase-activating protein.

## Competing interests

The authors declare that they have no competing interests.

## Authors' contributions

ESC drafted the manuscript. All authors analyzed the published data, read and approved the final manuscript.
